# Phytochemicals Approach for Developing Cancer Immunotherapeutics

**DOI:** 10.3389/fphar.2017.00386

**Published:** 2017-06-19

**Authors:** Shu-Yi Yin, Ning-Sun Yang, Tien-Jen Lin

**Affiliations:** ^1^Agricultural Biotechnology Research Center, Academia SinicaTaipei, Taiwan; ^2^Graduate Institute of Injury Prevention and Control, Taipei Medical UniversityTaipei, Taiwan; ^3^Department of Neurosurgery, Taipei Medical University-Wan Fang HospitalTaipei, Taiwan

**Keywords:** phytochemicals, herbal extract, cancer immunotherapy, tumor microenvironment

## Abstract

Phytochemicals or their derived compounds are being increasingly recognized as potentially potent complementary treatments for cancer. Among them, some phytochemicals are being actively evaluated for use as adjuvants in anticancer therapies. For instance, shikonin and hypericin were found to induce immunogenic cell death of specific cancer cells, and this effect was able to further activate the recognition activity of tumor cells by the host immune system. On the other hand, some derivatives of phytochemicals, such as dihydrobenzofuran lignan (Q2-3) have been found to induce the secretion of an endogenous anticancer factor, namely IL-25, from non-malignant cells. These findings suggest that phytochemicals or their derivatives confer a spectrum of different pharmacological activities, which contrasts with the current cytotoxic anticancer drugs commonly used in clinics. In this review, we have collected together pertinent information from recent studies about the biochemical and cellular mechanisms through which specific phytochemicals regulate target immune systems in defined tumor microenvironments. We have further highlighted the potential application of these immunotherapeutic modifiers in cell-based cancer vaccine systems. This knowledge provides useful technological support and know how for future applications of phytochemicals in cancer immunotherapy.

## Application of Phytochemical-Induced Immunogenic Cell Death in the Development of Cancer Immunotherapy

Immunogenic cell death (ICD) is a kind of cell death that can effectively stimulate specific immune response against dying or dead cell antigens (Kroemer et al., 2013). The molecular features of ICD has been characterized as specific expression changes in some DAMPs associated with the induced immunogenicity, for instance against targeted tumor cells. These immune-modifying molecules include heat shock proteins (HSP), HMGB1, CRT, glucose-related protein (GRP) and others (Garg et al., 2010; Krysko et al., 2012). As a result of these findings, the induction of tumor cell ICD and DAMP expression has been considered to be a useful therapeutic approach to activate the immunogenicity of target tumor cells.

Shikonin, a secondary plant metabolite isolated from *Lithospermum erythrorhizon*, can confer a broad spectrum of specific cellular and biochemical activities. These include the inhibition of promoter/transcriptional activities of the pro-inflammatory cytokines TNF-a (Staniforth et al., 2004) and GM-CSF (Su et al., 2008), the blockade of splicing of TNF-a pre-mRNA (Chiu and Yang, 2007), the induction of EMT activity in skin wound-healing (Yin et al., 2013a), and a differential effect on the genomic expression of cytokine/chemokine genes in human monocytes (Chiu et al., 2010). In our previous studies, shikonin was also found to strongly stimulate ICD of tumor cells (Chen et al., 2012), which induced a potent immune response through DCs to suppress tumor growth and/or metastasis (Lin et al., 2015a,b). A molecular target of shikonin, namely hnRNPA1, was proved to play an important role in shikonin-induced ICD in mammary tumor cells (Yin et al., 2016a). Moreover, hnRNPA1 was known as an important substrate of GzmA that can disrupt the nuclear export activity of newly synthesized RNA, and this resulted in a specific type of immune-mediated programmed cell death (Rajani et al., 2012). These findings together suggest that shikonin can confer various molecular or intracellular effects on the post-transcriptional processing of RNAs, including nuclear export and splicing activities of pre-mRNA. Importantly, the shikonin-caused dysfunction of hnRNPA1 may further provide a sound pharmacological basis for the potential application of shikonin in cancer immunotherapeutics, for example, in DC-based cancer vaccines.

Immunogenic cell death-associated immunogenicity will also be effective if it is induced by a specific ROS-based ER stress, such as hypericin-based PDT (Krysko et al., 2012), rather than by secondary or collateral ER stress effects. Hypericin, an anthraquinone derivative present in the yellow flower of *Hypericum perforatum* (Wurglics et al., 2001), has also been used to develop hypericin-based PDT (Zheng et al., 2014a). Recently, the use of DC vaccines in combination with hypericin-based PDP–induced ICD was applied to treat HGG in an animal model. ICD-based DC vaccines improved survival of test animals, and this effect was found to be dependent on cell-associated ROS and the release of DAMPs, including the extra-cellular HMGB1 and the surface CRT (Garg et al., 2016). These findings thus suggest that Hyp-PDT–based anticancer vaccines may also be worth development for clinical use in the future.

## Regulation of Immune Cells in the Tumor Microenvironment by Phytochemicals

The tumor microenvironment is a critical determinant of distant cancer metastasis (Mlecnik et al., 2016). Immune cells in the tumor microenvironment, including regulatory T cells, dendritic cells, MDSCs and TAMs, are known to express a low level of MHC class I molecules but high levels of various immunosuppressive factors, such as IL-10, IL-6, and TGF-β. And these factors can contribute to tumor progression and the evasion of systemic immune surveillance (Albini and Sporn, 2007). Currently, most anticancer drugs are based mainly on specific targeted or cytotoxic agents which were discovered by using the “one gene, one target, one disease” approach (Anighoro et al., 2014). Over the last few years, however, the application of some traditional medicines, such as GLPS extracted from *Ganoderma lucidum*, have been reported to enhance the CD4^+^/CD8^+^ T cell ratio in the tumor microenvironment (Liu and Sun, 2012; Sun et al., 2014). In addition, the anoectochilus formosanus and a schisandra polysaccharides isolated from *Schisandra chinensis*, were found to promote the M1 differentiation of TAM (Kuan et al., 2012). Recently, some plant natural products or phytoagents, including terpenoids, phenolics, plant-derived lipids, alkaloids and PHY906 (a multi-herb formulation), have also highlighted and summarized for their effects on the oxylipin dynamics in a defined tumor microenvironment (Apaya et al., 2016). These studies on natural compounds have brought into clarity the poly-pharmacological nature of phyto agents, which can be thought of as “multiple targets, multiple effect and complex disease”.

Myeloid-derived suppressor cells (MDSCs) have been found to be largely responsible for the inhibition of host antitumor immunities, consequently impairing the efficacy of anticancer immunotherapeutic approaches (Gabrilovich and Nagaraj, 2009). Some phytochemicals or herbal extracts have been shown to confer suppressive activity in controlling MDSC expansion. Silibinin, a flavanone from the seeds of *Silybum marianum*, is a dietary supplement which is widely used for hepato- and chemo-preventive activities (Agarwal et al., 2006). Among several effects of silibinin in the tumor microenvironment (Deep and Agarwal, 2013), the supplement was found to strongly suppress the formation of 5-LOX metabolites in human macrophages, mast cells and granulocytes, and inhibit COX-2 expression *in vivo* (Tager et al., 2001; Bannwart et al., 2010). In a murine mammary tumor model, silibinin treatment also inhibited the accumulation of MDSCs, increased the quantity of T cells in the tumor-associated microenvironment and increased the survival rate of test mice (Forghani et al., 2014). Recently, an ethanol extract of *Bidens pilosa* (BP-E) was found to effectively suppress the G-CSF-induced differentiation of gMDSCs from mouse myeloid precursor cells. Consistently, *in vivo* studies further demonstrated that oral delivery of BP-E can effectively suppress 4T1 tumor metastasis in a tumor-resection model (Wei et al., 2016). Our bio-organic chemical analysis also showed that a specific group of polyacetylenic glycosides may act as the principle phytochemicals responsible for the detected MDSC activities, *ex vivo* and *in vivo* (Wei et al., 2016). These findings clearly indicate that specific types of polyacetylene may have therapeutic potential against the metastasis of mammary tumor cells.

In our previous studies, plant extracts of different plant species were shown to actively enhance the immunity or efficacy of a DC-based vaccine against tumor metastasis in animal models (Chang et al., 2013, 2015). A specific, ethanol-precipitated fraction of *Dioscorea alata* var. *purpurea* Tainung No. 5 extract, designated as DsII-TN5, effectively enhanced the DC-mediated activation of T-cell proliferation and suppressed the growth of melanoma tumor cells *in vivo* (Chang et al., 2013). Mechanistically, other studies showed that immunological activities of specific polysaccharides and/or glycoproteins in Dioscorea tubers were in part due to their TLR4-signaling pathway-mediated immunomodulatory and cytokine-regulation activities (Fu et al., 2006; Liu et al., 2008). Moreover, our previous study further showed that Dioscorea phytoextracts can enhance the proliferation of murine splenocytes *ex vivo* and promote regeneration of specific myeloid-derived progenitor cells in chemotherapy-damaged bone marrow tissues (Su et al., 2011). These findings suggest that certain plant polysaccharides and/or glycoproteins in Dioscorea extracts may serve as potential candidates for acting as non-toxic TLR4 agonists that can activate TLR4 signaling to induce anti-tumor immune responses. Other traditional Chinese medicine preparations, including root extracts of Radix Astragalus (*Astragalus membranaceus*) and Radix Codonopsis (*Codonopsis pilosulae*), were also shown to modulate the functions of specific immune cells. In particular, the highly purified *Astragalus* polysaccharides can activate mouse macrophages and B cells (Shao et al., 2004), restore depressed mitogen response and inhibit the growth of leukemia and lymphoma tumor cells in tumor-bearing mice (Cho and Leung, 2007). In addition, other studies also indicated that the *Codonopsis* polysaccharides caused a change in T-cell polarization from Th2 to Th1 responses and further suppressed Treg cells (Zheng et al., 2014b). Clinically, these polysaccharides may be responsible for conferring pharmacological bioactivities for repair or restoration of immunosuppressive activities in treated patients (Wang et al., 2012). To similarly address this issue, in our previous study, we further evaluated two highly purified but still mixed polysaccharide fractions from the root of *A. membranaceus* (Am) and *Codonopsis pilosulae* (Cp), for their use in stimulating a highly anti-metastatic efficiency of a DC-based cancer vaccine against 4T1 mammary carcinoma *in vivo* (Chang et al., 2015). Our findings suggest that these plant polysaccharides in Am and Cp can also be effectively employed as a potent adjuvant for development of DC-based cancer immunotherapies.

## Suppression of Oxidative Stress in the Tumor Microenvironment by Phytochemicals

Reactive oxygen species (ROS), including superoxide, hydroxyl radicals or non-reactive oxygen, are a group of molecules that contain reactive oxygen and can be readily converted into a reactive species, such as H_2_O_2_ (Son et al., 2013). In the tumor microenvironment, ROS have been observed to play multiple roles in the hallmarks of carcinogenesis, such as genome instability and mutation, angiogenesis, invasion and metastasis (Grivennikov et al., 2010). Several phytochemicals or herbal extracts have been evaluated in clinical trials as well as in many studies for their anti-cancer activities through the modulation of oxidative stress and the tumor microenvironment. Among them, the proposed pharmacological mechanisms of selected phytochemicals or herbal extracts, such as curcumin, resveratrol, artemisinin, berberine, paclitaxel, broccoli isothiocyanate, green tea extract, mistletoe extract, noscapine and its derivatives and piperine have been evaluated in clinical trials, as well as intensively studied in preclinical mouse tumor models for their anti-cancer activities via the modulation of oxidative stress in the tumor-associated microenvironment (Cheng et al., 2016). These pleiotropic functions of phytomedicines or phytoagents have been considered to be a novel adjuvant approach that may be useful in combination with chemotherapeutics for overcoming drug resistance or tumor metastasis in cancer therapy (Cheng et al., 2016).

## Potential Pharmacological Activities of Phytochemicals in Activating the Expression/Secretion of Specific Endogenous Anticancer Factors in the Tumor Microenvironment

Tumor suppressor p53 is a transcription factor that can play important roles in the regulation of the DNA repair, cell-cycle, apoptosis, angiogenesis and senescence (Shangary and Wang, 2008). Through activation of p53, many herbal extracts, including the extracts of *Hibiscus syriacus* (Cheng et al., 2008), *Bing De Ling* (Xu et al., 2005), *Suillus collinitus* (Vaz et al., 2012), *Emilia sonchifolia* (Lan et al., 2012), *Cochinchina momordica* (Liu H.R. et al., 2012), Zeng Sheng Ping (Zhang et al., 2004), and JP-1 (Yao et al., 2016), have been shown to induce growth arrest or metastatic activity of test cancer cells. These herbal medicines or their derived compounds have been contemplated as potential resources in the search for proper agents to restore the p53 function in cancer cells. Mechanistically, p53 can also further stimulate the expression of specific sets of target microRNAs, such as miR-34a (He et al., 2007), and this is known to play an essential role in the anti-proliferative activities of specific phytochemicals, such as artemisinin and artesunate (Hargraves et al., 2016).

Mutations or deletions in the human p53 gene (*TP53*) are present in nearly 50% of human cancers (Wang et al., 2017). One p53-inhibitory protein, namely murine double minute 2 (MDM2), tightly controls the expression levels of p53 protein (Klein and Vassilev, 2004). Mechanistically, MDM2 induces degradation of p53 via the ubiquitin-proteasome machinery through the binding and blocks to the N-terminal transactivation domain of p53 (Klein and Vassilev, 2004). The crystal structures of MDM2 and p53 further indicate their interaction is mediated by a surface pocket and three hydrophobic key residues: Phe19, Trp23, and Leu26 (Wiman, 2010). Because of the antagonistic role of MDM2 in controlling p53 expression, many small molecules have been designed or developed to mimic p53-binding residues (Okabe et al., 2008). Among them, some herbal compounds, such as α-mangostin and gambogic acid, were indicated to inhibit the p53-MDM2 interaction by binding to MDM2 (Parks et al., 2005; Hientz et al., 2017). These two compounds exhibited high binding affinities with hydrophobic MDM2 through the residues Gly58, Asp68, Val75, and Cys77 (Parks et al., 2005). These findings suggest that specific phytochemicals may also be valuable contenders in cancer therapeutics by targeting the MDM2-p53 interaction.

Specific tumor microenvironment systems are also associated with different dynamic feedback systems for secretion of some cytokines, endogenous second messengers or other soluble factors between cancer and the surrounding stromal cells (Swartz and Lund, 2012). Some tumor-associated stromal cells can produce tumor suppressor factors, such as nucleoside NME1 (Dooley et al., 1994), Kangai 1 (KAI1/CD82) (Dong et al., 1997) and IL-25 (Furuta et al., 2011), in the tumor microenvironment, and these activities can restrict the development or metastasis of surrounding tumor cells. The investigation of specific molecular agents that can confer stimulatory effect on the expression or secretion of these endogenous anticancer factors is hence a new direction that may lead to new cancer therapies (Bykov et al., 2002). Among these endogenous anticancer factors, IL-25 (IL-17E) secreted by normal mammary epithelial calls has been reported to confer a specific anticancer effect on breast cancer cells, however, it has little or no cytotoxic effect on the non-malignant counterpart cells (Furuta et al., 2011). This apoptosis-promoting activity of IL-25 may be due to the differential expression of its receptor, IL-25RB. The expression level IL-25RB was found to be much higher in tumors from patients with poor prognoses, as comparing to that in non-malignant breast tissues (Furuta et al., 2011). Recently, our study showed that a dimerization product of plant caffeic acid methyl ester, the dihydrobenzofuran lignan (Q2-3), can effectively suppress the metastasis of mammary tumor cells (Yin et al., 2016b). By using a tumor resection model, we demonstrated that the *in vivo* treatment of Q2-3 efficiently induced the TAFs to secrete IL-25 at high levels. This IL-25 secretion was shown to play a key role in the Q2-3-mediated anti-metastatic activity in mouse mammary tumor models. In addition, our *in vivo* experiments further showed that the anti-metastatic effects of Q2-3 on both mouse (4T1) and human (MDA-MD-231) tumor cells are additive, when it was administrated in combination with the clinically used anticancer drug, docetaxel (Yin et al., 2016b). Together, our findings revealed that the secretion of IL-25 from TAFs could serve as a highly inducible therapeutic strategy for the control of mammary tumor metastasis. Moreover, our study again indicated that the potential pharmacological activity of specific phytochemicals or their synthetic compounds may effectively activate the secretion/expression of specific endogenous anticancer factors in the tumor microenvironment, resulting in strong anti-metastatic effect.

## Summary and Future Prospects

**FIGURE 1:**
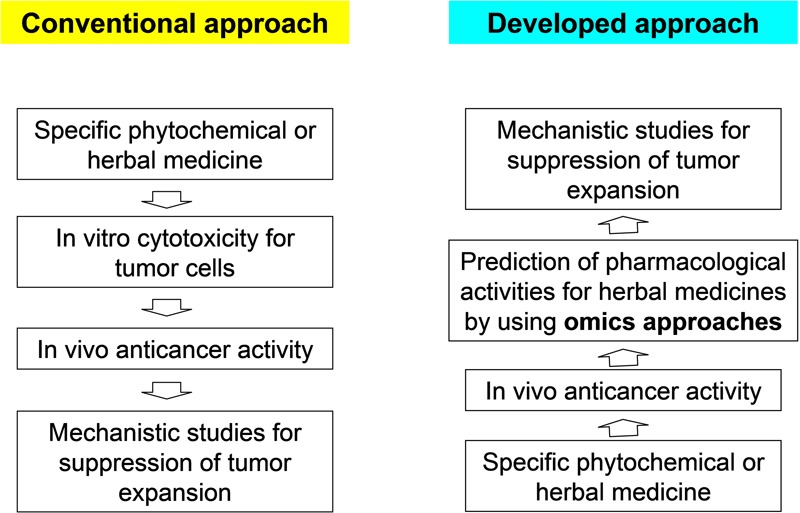
Omics approaches to reveal the *in vivo* anticancer activity of herbal edicines. According to the conventional approach, studies are initiated by screening the *in vitro* cytotoxicity of tumor cells. In this way, it can be efficiently determined which compound or active component in an herbal medicine can confer a cytotoxic effect on cancer cells. To overcome the limitation of this approach when studying issues related to immune regulation or other cellular activities in the tumor microenvironment, using the developed approach, studies can be initiated through a combination of omics approaches and different *in vivo* tumor model systems. Other mechanistic studies for suppression of tumor expansion can be used to confirm the hypothesis formulated through the omics analyses.

Although different pharmacological activities of phytochemicals or herbal extracts have been indicated for the development of cancer immunotherapies (**Table 1**), the exact molecular/cellular targets and hierarchical mechanisms for many of them still need to be evaluated. To address issues related to immune regulation or non-malignant cells in the tumor-associated microenvironment, we consider that new strategies that combine specific *in vivo* tumor model systems with various omics tools/approaches, as shown in **Figure 1**, may provide new directions for phytomedicinal research. Conventional phytomedicines are thought to involve multiple active components and interact with multiple targeted molecules related to various cellular or physiological effects (e.g., immunomodulation). Through considering different responsive elements for their observed medicinal efficacy, including the expressional change of genes, proteins, microRNA and other metabolites, we propose a hypothesis or a prediction for specific pharmacological activity of phytochemicals after systematic analysis (**Figure 1**). Although many herbal medicines are used worldwide, their efficacies for disease control or health care, have unfortunately only been studied in a sporadic way, with few or no RCTs (Yin et al., 2013b). In 2004 an international group of pharmacologists, methodologists, pharmacognosists, and clinical trial researchers met for a consensus-forming meeting, which develop the recommendations for the reporting of herbal medicine trials in Toronto, Canada (Gagnier et al., 2006b). These recommendations and CONSORT statements aimed to assist researchers to accurately assess the reproducibility of herbal medicine trials and internal/external validity, to allow the safety and efficacy of specific herbal medicines be assessed in a more accurate way (Gagnier et al., 2006a). The 22 CONSORT checklist items including title and abstract, background, participants, outcomes, baseline data, interpretation, generalizability, detailed recommendations for interventions, and the overall evidence (Gagnier et al., 2006b). For future clinical applications of specific herbal extracts or phytochemicals in cancer immunotherapy, we expect more stringent RCTs to be performed after systematic and modern biology-defined preclinical studies have determined particular formulas/phytochemicals to be promising.

**Table 1:** Different pharmacological activities of phytochemicals or medicinal herb extracts that are promising candidates for the development of cancer immunotherapeutics.

Strategies for cancer immunotherapy	Specific phytochemicals or herbal extracts	Specific effect on targeted cancer or *in vivo* tumor models	Reference
(1) Induction of immunogenic cell death for enhancing efficacy of tumor vaccines	Shikonin	B16 melanoma or 4T1 mammary carcinoma mice tumor model	Chen et al., 2012; Lin et al., 2015b; Yin et al., 2016a
	Hypericin	Orthotopic high grade glioma (HGG) mice tumor model	Krysko et al., 2012
(2) Activation of specific immune cell types or immunity for cell-based vaccine potency in the tumor microenvironment	Polysaccharides (GLPS) from *Ganoderma lucidum* *Anoectochilus formosanus* and schisandra polysaccharide isolated from *Schisandra chinensis*	Plasma-induced suppression of lymphocyte activation in lung cancer patient	Liu Q. et al., 2012; Sun et al., 2014 Kuan et al., 2012
	*Dioscorea alata* var. *purpurea* extract (DsII-TN5)	B16 melanoma mice tumor model	Chang et al., 2013
	Extracts of *Astragalus membranaceus* and *Codonopsis pilosulae*	4T1 mammary carcinoma mice tumor model	Shao et al., 2004; Chang et al., 2015
(3) Suppression of gMDSC activity	Silibinin	The suppressive effects of silibinin on progressive brain metastases in non-small cell lung cancer patients 4T1 mammary carcinoma mice tumor model	Bosch-Barrera et al., 2016 Agarwal et al., 2006
	Extract of *Bidens pilosa* (BP-E)	4T1 mammary carcinoma mice tumor model	Wei et al., 2016
(4) Regulation of oxylipin dynamics	Monogalactosyl-diacyl-glycerols (MGDG)	B16 melanoma mice tumor model	Hou et al., 2007;Apaya et al., 2016
(5) Suppression of oxidative stress	Curcumin, resveratrol, artemisinin, berberine, paclitaxel, broccoli isothiocyanate, green tea extract, mistletoe extract, noscapine and its derivatives, and piperine		Related references as summarized, (Cheng et al., 2016)
(6) Induction of endogenous anticancer factors (p53 or IL-25)	Extracts of *Hibiscus syriacus*	A549-xenograft (human lung cancer) mice tumor model	Cheng et al., 2008
	Bing De Ling	CT26-xenograft (human cancer cell line) mice tumor model	Xu et al., 2005; Zhang et al., 2010
	Extracts of *Suillus collinitus*		Vaz et al., 2012
	Extracts of *Emilia sonchifolia*	B16 melanoma mice tumor model	Lan et al., 2012
	Extracts of *Cochinchina momordica*		Liu H.R. et al., 2012
	Zeng Sheng Ping	A/J mice (dominant-negative *p53* mutation and/or a heterozygous deletion of *Ink4a/Arf*) model for human lung adenocarcinoma cells	Zhang et al., 2004
	JP-1		Yao et al., 2016
	Artemisinin and artesunate	The phase I trial defines a well-tolerated dose of oral artesunate (200 mg/d) in patients with metastatic breast cancer	Hargraves et al., 2016; von Hagens et al., 2017
	Dihydrobenzofuran lignan (Q2-3)	4T1 mammary carcinoma tumor model	Yin et al., 2016b

## Author Contributions

All authors listed have made a substantial, direct and intellectual contribution to the work, and approved it for publication.

## Conflict of Interest Statement

The authors declare that the research was conducted in the absence of any commercial or financial relationships that could be construed as a potential conflict of interest.

## Abbreviations

CRT: calreticulin
DAMPs: damage-associated molecular patterns
DCs: dendritic cells
EMT: epithelial-to-mesenchymal transition
ER: endoplasmic reticulum
GLPS: *Ganoderma lucidum* polysaccharides
H_2_O_2_: hydrogen peroxide
HGG: high-grade glioma
HMGB1: high-mobility group box 1 protein
hnRNPA1: heterogeneous nuclear ribonucleoprotein A1
ICD: immunogenic cell death
ITC: isothermal titration calorimetry
KAI1/CD82: Kangai 1
MDSC: myeloid derived suppressor cell
NME1: diphosphate kinase A
PKM2: pyruvate kinase-M2
PDT: photodynamic therapy
RCTs: randomized and controlled trials
ROS: reactive oxygen species
SK: shikonin
TAF: tumor-associated fibroblast
TAM: tumor-associated macrophage
TCL: tumor cell lysate
TNF-α: tumor necrosis factor-α
